# Visual Locating of Reactor in an Industrial Environment Using the Composite Method

**DOI:** 10.3390/s20020504

**Published:** 2020-01-16

**Authors:** Chenguang Cao, Qi Ouyang, Jiamu Hou, Liming Zhao

**Affiliations:** 1 School of Automation, Chongqing University, Chongqing 400044, China; guangcc@foxmail.com (C.C.); SWPUhjm@163.com (J.H.); 2 School of Advanced Manufacturing Engineering, Chongqing University of Posts and Telecommunications, Chongqing 400044, China; zhaolm@cqupt.edu.cn

**Keywords:** sherardizing, reactor, YOLO, handling-hole, Hough transform

## Abstract

To achieve an automatic unloading of a reactor during the sherardizing process, it is necessary to calculate the pose and position of the reactors in an industrial environment with various amounts of luminance and floating dust. In this study, the defects of classic image processing methods and deep learning methods used for locating the reactors are first analyzed. Next, an improved You Only Look Once(YOLO) model is employed to find the region of interest of the handling hole and a handling hole corner detection method based on the image morphology and a Hough transform is presented. Finally, the position and pose of the reactors will be obtained by establishing a 3D handling hole model according to the principle of a binocular stereo system. To test the performance of the proposed method, a set of experimental systems was set up and experiments were conducted. The results indicate that the proposed location method is effective and the precision of the position recognition can be controlled to within 4.64 mm and 1.68° when the cameras are approximately 5 m away from the reactor, meeting the requirements.

## 1. Introduction

Sherardizing is an important method for the formation of corrosion-resistant Fe-Zn layers on steel [1]. The corrosion resistance of steel is significantly improved after this method, which has been widely used in steel workpieces. However, sherardizing has a low-level automation owing to its complicated technology, and there are steps that need to be conducted manually. This is not only inefficient, but is also bad for worker health, the reason for which is that some zinc powder will inevitably be inhaled by workers during long-term work even if wearing a dust mask. Therefore, it is necessary to intelligentize the industry.

**Figure 1:**
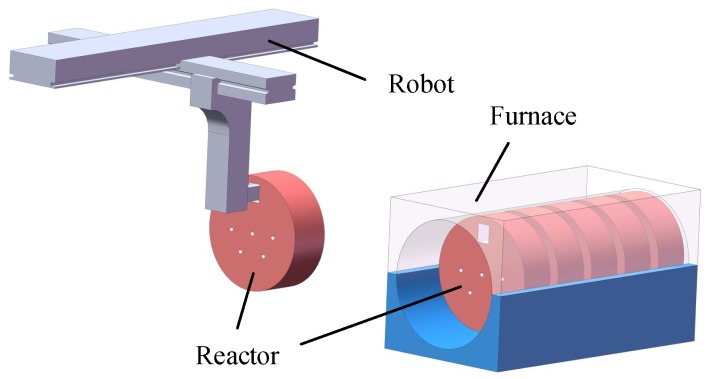
Schematic of a new automatic unloading system.

Figure 1 shows a schematic of the automatic unloading during the sherardizing. These reactors are used to hold steel workpieces and zinc powder, and a square handling hole and numerous circular vent holes are present at the bottom of the reactors. During the heating process, the reactors continuously rotate with the furnace and thus the steel workpieces will be covered in zinc powder. After heating, the furnace needs to continue to rotate to place the outermost reactor near the furnace door in an ideal pose. The reactor can then be transported easily by a robot, as shown in Figure 1. At present, the locating of the reactors is conducted manually. To achieve an automatic uploading, a sensor must be used in place of a human worker to obtain the pose and position of the reactors, which is the core part of the automatic uploading system. If a camera is used as the sensor and image processing methods are applied to obtain the location information from an image, the above process can be considered a visual location in an industrial environment. It should be noted that the concept of a visual location is quite different between the fields of mobile robot navigation and industry. The former aims to find the position of a robot in the environment [2]. For the latter, the goal is to calculate the pose and position of the object in the reference coordinate by applying image processing and spatial geometry. These are clearly different, and thus the concept of visual location here refers to the locating of an object.

The visual location system is similar to a visual measurement system [3] and a visual detection system [4], and has a higher efficiency and lower error rate than a manual operation. In addition, a visual location system is often used in conjunction with robots. A robot system based on visual location has significant value and has been successfully applied to an intelligent assembly [5], automatic welding [6], agricultural picking [7], and robot sorting [8]. To maximize the advantages of this system, the efficiency and precision are two indispensable requirements for a vision location system. However, these requirements could not be achieved at the same time. In other words, there are many requisite steps that are needed when we are pursuing a higher location accuracy, which will increase the time consumption. This indicates that some simple and effective image processing methods should be used in a vision location system. For example, a circular projection [9], Hu moment [10], and various template matching methods are employed to find different objects. The area, center, and orientation are three of the most commonly used parameters in visual location, and are calculated using the image moments [11]. Although these methods have been proven to be effective, an ideal working environment is needed, which is difficult to achieve in some factories. The reason is that images obtained in industrial environments are not perfect. The commonly used image segmentation methods are hard to separate the object and background. In this case, the position and pose could not be calculated accurately by these simple image processing methods owing to the poor robustness. Therefore, the scope of application of the visual location system is greatly limited.

Fortunately, the purposes of visual location methods and object detection methods are the same, both aiming to find objects in an image. This also provides another method for visual location. It is widely accepted that the progress of object detection has generally passed through two historical periods: a traditional object detection period and a deep-learning-based detection period [12]. Compared with a traditional detection method, the deep learning detection method makes the recognition of an object in a complex environment possible owing to its extraction of the high-dimensional features of the object. To achieve the practicality of this method, numerous detection models have been proposed. Initially, several two-stage detection models including R-CNN [13], fast R-CNN [14], and a faster R-CNN [15] were proposed. These models have advantages of a high precision and strong generalization, although the time consumption is unsatisfactory because large amounts of computing resources are consumed by a regional suggestion [16], and the requirement of a real-time processing has yet to be met. Shortly thereafter, single-stage detection models represented by a Single Shot MultiBox Detector (SSD) [17] and the You Only Look Once (YOLO) [18] method were proposed. These two models regard object detection as a regression problem, and some advanced strategies, such as anchor [15,19], bounding box regression [13,14,15,17,18,19,20], and multi-scale prediction [20], have been introduced. Therefore, the efficiency of a single-stage detection method is extremely high and they have shown superiority in object detection and have been applied in numerous fields in recent years [21]. As one of the most representative networks, the YOLO network can get good performance in both speed and accuracy. In CVPR2017, YOLOv2 was presented and YOLOv3 was presented in 2018. The state-of-the-art version is not only faster and more accurate than the previous version, but also performs well with detecting small targets.

It should be noted that, although many methods are effective, they are unsuitable for industry for which location accuracy should be within several millimeters. The calculation result of advanced object detection methods based on deep learning is a rectangular box containing the object rather than a coordinate. The reason for this lies in the difference of concept between the object detection methods and classical visual location methods. For object detection based on deep learning, we need to mark the pedestrians by red rectangular boxes and make sure there are no omissions. The box is worthless for industrial location and there is no doubt that the location error is huge if the midpoint of the box is used as the result. This is difficult to achieve through an object detection method based on deep learning solely, and thus such methods have been limited in industrial applications.

For the automatic unloading system in Figure 1, it works in a sherardizing factory that is filled with dust, which cannot be completely removed by the air circulation system. The effect of dust on an image is similar to fog, both making the boundary between the object and adjacent area unclear. The other obvious reason is an unstable luminance. The luminance must be different in the factory between the day and night, even if an auxiliary light is used. According to the above analysis, most existing methods are not suitable for this system. In this study, a new binocular visual location method suitable for locating a reactor is presented. This method combines traditional location methods with the YOLO model, and the reactor location is realized by calculating the pose and position of the handing hole. The problems caused by dust and unstable light cannot be solved, and an accurate locating of the reactors can be achieved using this method. The rest of this paper is organized as follows. Section 2 establishes a binocular visual location system. Section 3 clarifies the limitations of existing methods and describes a new visual location method in detail. Section 4 introduces the relevant experiments and discusses the experiment results. Finally, some concluding remarks are provided in Section 5.

## 2. System Design and Calibration

### 2.1. System Structure

**Figure 2:**
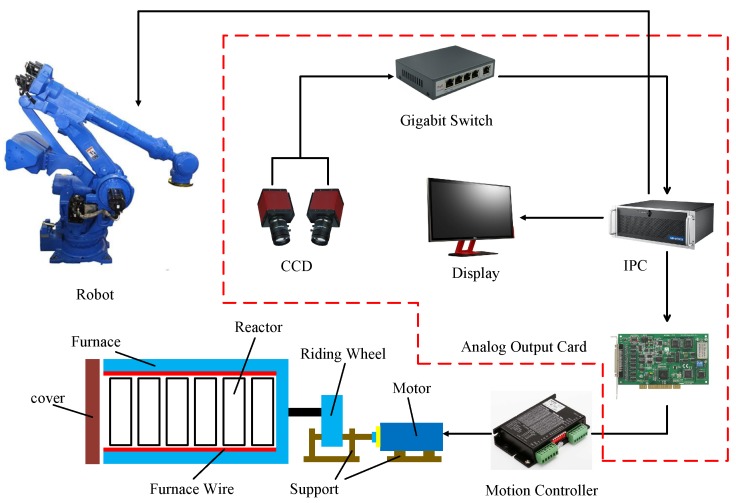
Structure of the automatic unloading system.

An automatic unloading system based on binocular vision is shown in Figure 2 and the visual location system is marked in the red dotted box. The visual location system consists of two CCDs, a gigabit switch, an industrial personal computer (IPC), and an analog output card with a peripheral component interconnect (PCI).

The system works as follows: First, the images are captured in real time by the CCD. Next, the position and pose of the handing hole will be calculated using the proposed visual location method. This part is the core procedure, which determines the accuracy and efficiency of the system. There are two points that should be noticed. The first one is that the handing hole rather than the whole reactor is regarded as a locating object. The reason is that the handing-hole is a big rectangular hole on the reaction surface and the difference between the handing hole and the surrounding is obvious. It is convenient to process the handing-hole rather than the whole reactor. The second one is that the locating result could be shown as a locating vector (z,θ). *Z* is used to describe the position of the reactors because there are two movement directions of reactor in the furnace. θ is used to describe the pose of reactors. Finally, the data are output. An analog output card is then used to convert it into two current signals for which a range is 5–30 mA. These signals are regarded as the input of the motion control system used to adjust the state of the reactors.

**Figure 3:**
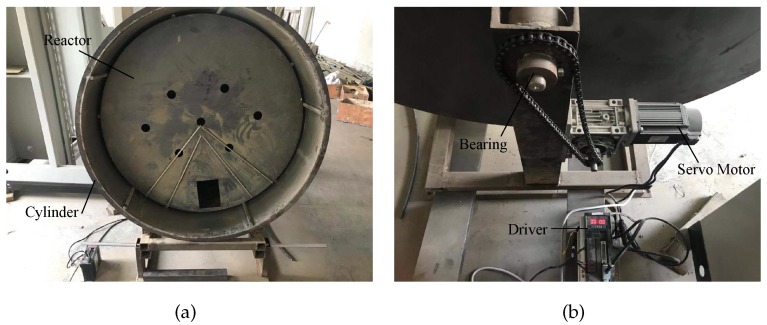
Experimental model. (**a**) front of the model; (**b**) back of the model.

At present, a sherardizing factory is still under construction. To verify the effectiveness of the visual location system, a rotatable furnace model is produced, as shown in Figure 3. The experimental model consists of a rotatable cylinder, a disk, and a servo motor for simulating the furnace, reactor, and dynamical system, respectively. The cylinder is rotated by the motor, which rotates the disc through friction, and thus a real situation can be simulated. The size of the disk is the same as that of the reactor, the diameter of which is 1200 mm and the size of the handing-hole is 180 mm × 130 mm. There are many round holes on the surface of the disk simulating the vent holes.

### 2.2. Camera Selection and Calibration

The requirements should be analyzed before the equipment selection because the equipment parameters are related to the accuracy of the system. According to the planning scheme, we need to trace the handing hole in the entire surface of the reactor, and the location error should not exceed 6 mm and 2°. The CCD should be installed on the side with at least a 4 m distance because this area will be occupied by a robot and the transport vehicles. To meet the accuracy requirements, the real size corresponding to a single pixel is no more than 1 mm. According to the test, an MV-EM200M model CCD is selected with a 1600×1200 pixel resolution and a 25 mm lens.

A camera calibration is an important step in 3D computer vision for extracting metric information from 2D images. In this paper, we need to correct the lens distortion and epipolar line and acquire the camera parameters through a calibration process. The pinhole model can be used to describe the principle of the CCD as follows:

(1) $$Z_{c}\left(\begin{array}{c} u\\ v\\ 1 \end{array}\right)=\left(\begin{array}{ccc} \frac{f}{dx} & 0 & u_{0}\\ 0 & \frac{f}{dy} & v_{0}\\ 0 & 0 & 1 \end{array}\right)\left(\begin{array}{cc} R & T \end{array}\right)\left(\begin{array}{c} X_{w}\\ Y_{w}\\ Z_{w}\\ 1 \end{array}\right),$$

where *f* is the focal length, dx and dy are the distances between adjacent pixels in the *u* and *v* axes, (u,v) is the pixel coordinate, $\left(X_{w},Y_{w},Z_{w}\right)$ is the world coordinate, and (R,T) is the transfer matrix.

A lens distortion always occurs owing to manufacturing errors of the lens and a deviation in the assembly. This unwelcome factor is manifested by the deviation between the ideal pixel coordinates $\left(u_{i},v_{i}\right)$ and the actual pixel coordinates $\left(u_{a},v_{a}\right)$. The relationship between these two coordinates is as follows:

(2) $$\left\{\begin{array}{c} u_{i}=k_{1}u_{a}r_{a}+k_{2}u_{a}r_{a}^{2}+p_{1}\left(2u_{a}^{2}+r_{a}^{2}\right)+2p_{2}u_{a}v_{a},\\ v_{i}=k_{1}v_{a}r_{a}+k_{2}v_{a}r_{a}^{2}+p_{2}\left(2v_{a}^{2}+r_{a}^{2}\right)+2p_{1}u_{a}v_{a}, \end{array}\right.$$

where p1, p2, and p3 are the radial distortion coefficients, and k1 and k2 are the tangential distortion coefficients. All camera parameters can be calculated using the method developed by Zhang [22]. This method overcomes the defect of the high-precision calibration required by a traditional calibration method, and the result is more accurate than a self-calibration method [23]. Therefore, the Zhang calibration method is widely used in machine vision. The calibration board has nine divisions, each of which is 30 mm × 30 mm in size. The calibration board is generated using OpenCV and then printed using a high-precision printer.

The purpose of epipolar line correction is to adjust the distortion caused by the camera pose. After a correction, the optical axes of the two CCDs are parallel and the height of one point on both the left and right images is the same. Therefore, only the matching points in the same row are searched, which can greatly improve the efficiency of the stereo matching.

## 3. Deficiency of the Existing Visual Method in Handling Hole Location

### 3.1. Deficiency of Traditional Location Method

The contour is often used to find an object and calculate the center coordinate when using a traditional visual location method. This means we should first separate the object from the image, which is called image segmentation. The core step of image segmentation is to find a range $\overset{˚}{U}\left(\theta_{thresh}^{n}\right)$ that sets up Equation (3):

(3) $$f\left(x,y\right)=\left\{\begin{array}{c} object,\;f\left(x,y\right)\in \overset{˚}{U}\left(\theta_{thresh}^{n}\right),\\ background,\;other, \end{array}\right.$$

where $\overset{˚}{U}$ is related to several threshold $\theta_{thresh}$. It is difficult to find a valid $\overset{˚}{U}\left(\theta_{thresh}^{n}\right)$ for some complicated images to separate objects.

**Figure 4:**
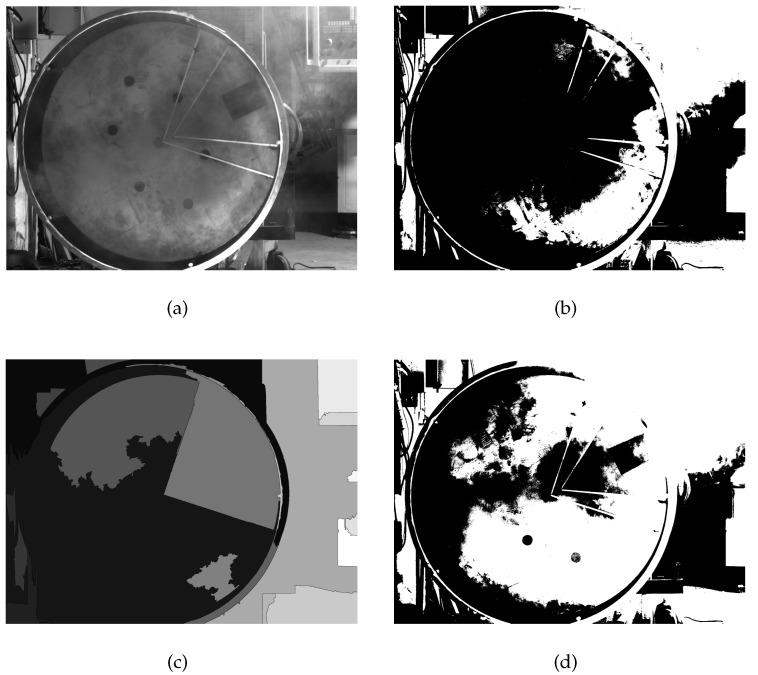
The results of classical image segmentation. (**a**) raw image; (**b**) Otus; (**c**) watershed algorithm; (**d**) histogram segmentation

Figure 4a shows a raw image with dust, and the other three images are the results obtained using Otus [24], a watershed algorithm [25], and histogram segmentation, respectively. The results indicate that, although these methods have been proven to be effective in other fields, they have difficulty separating handling holes from an image. Through an experimental analysis, there are two main factors regarding this case. The first is unwelcome dust. In an ideal environment, the gray-scale value of a handling hole is lower than the background, and it is easy to separate the object through a threshold segmentation. Dust can be regarded as numerous solid opaque particles that may block or change the propagation path of the light. The contrast between the handling hole and background is reduced by dust, creating an inconsistent contrast. It is difficult to find a $\overset{˚}{U}\left(\theta_{thresh}^{n}\right)$ that can be applied in all images. The second factor is inconsistent light, which is a classic problem for image segmentation. To achieve an uninterrupted production, it is necessary to consider the difference in light between day and night. The pixel distribution of the image is not the same under different light conditions, even if the object can be distinguished. This is extremely problematic for a pixel-based segmentation method. As mentioned above, such classic image segmentation methods are invalid for this type of case.

### 3.2. Deficiency of the Deep Learning Method

**Figure 5:**
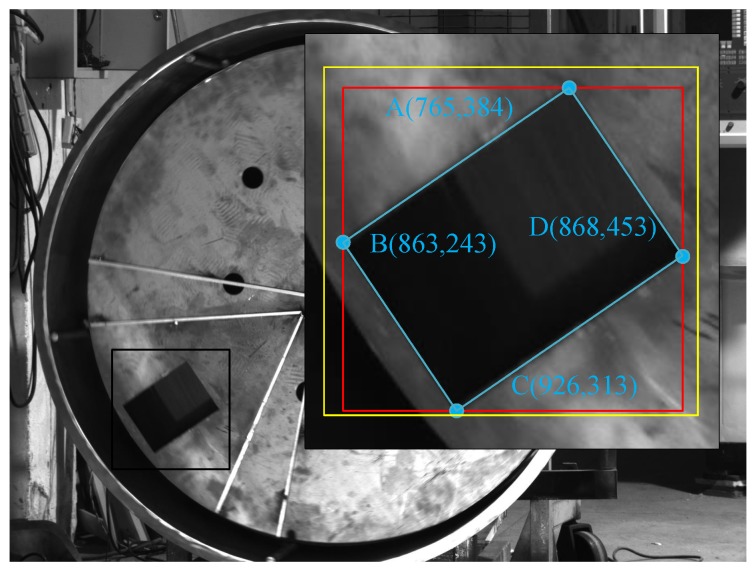
The MBR and counter of the handing-hole

In this section, we discuss the limitations of deep learning methods in a handing hole location. Before furthering the discussion, the concept of a minimum bounding rectangle (MBR) should first be introduced. An MBR refers to the smallest of boxes that can completely contain an object. This is an ideal box rectangle and is difficult to find. By contrast, a ground-truth bounding box refers to a rectangular box that can completely contain an object. This type of rectangle box is made through a manual operation. During the annotation, we try to make the ground-truth bounding boxes close to the MBR. There is a deep implication regarding the above description. Ground-truth bounding boxes made by different people are not exactly the same, and such boxes are usually slightly bigger than an MBR to allow the object to be completely contained. Figure 5 shows a schematic in which the yellow box is a ground-truth bounding box and the red box is an MBR. It is clear that there is a difference between the MBR and the ground-truth bounding box.

One primary goal is to find the accurate centers of the handling holes in the images. The MBR center of a handling hole must be the center of the handling hole because such a hole is rectangular. In theory, we can find the MBR of an object using a deep learning method to achieve its location. However, a deviation will occur between the network output and the MBR because the annotation boxes used in training are not an MBR. The center of a predicted bounding box is not the center of a handling hole, the reason for which is obvious in that an intersection over union (IoU) is used to express the accuracy of the network in the training, and the final value of the IoU is generally greater than 0.9, rather than 1. Assuming that the size of a predicted bounding box is 200×200 and there are 4000 pixels, an error will occur owing to a 0.1 deviation in the IoU. This is a major measurement error, and little improvement can be obtained by increasing the number of training steps and improving the quality of the annotation.

In addition, the part caused by the perspective projection can be called a minor error. This error is caused by the non-perpendicularity between the optical axis and the surface of the reactor. In this case, the shape of the handing hole in the image is a quadrilateral rather than a perfect rectangle. In Figure 5, the blue lines indicate a counter of the handling hole, and AB and CD should in theory be equal to BC and AD, respectively, but are not the same in actuality. Thus, we cannot prove that the center of the MBR coincides with the center of the handling hole.

Another goal is to obtain the pose of the reactor by calculating the rotation angle of the handling hole. There is one handing hole and several small vent holes in the surface of reactor according to the planning, and it is difficult to find another obvious object. In this case, it is difficult to calculate the rotation angle using a prediction box.

## 4. Method for Handling Hole Location in an Industrial Environment

**Figure 6:**
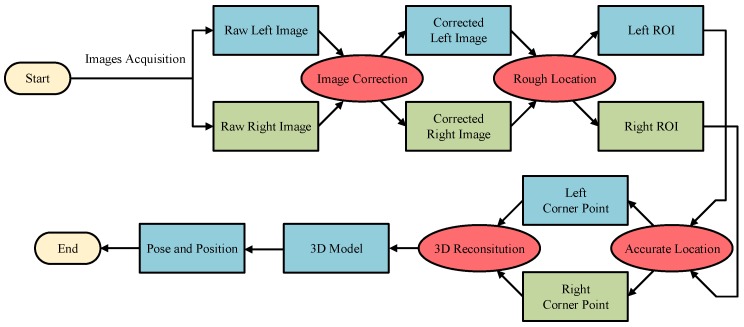
Flowchart of the RAL method.

It can be seen from the above analysis that, although the deep learning method can find an object in a complex and varied environment, it is difficult to achieve our goals. By contrast, the traditional method has a good effect with a poor robustness. Therefore, a novel roughly accurate location (RAL) method, which is divided into three parts, is presented to find the position and pose of the handling hole in an industrial environment. The method applies a complex technique integrating a YOLO model, Hough transform, and 3D reconstruction. The details of this method are illustrated below, and a flow chart is shown in Figure 6.

### 4.1. Rough Location Based on YOLO-MobileNet

The rough location is used to find the region of interest (ROI), which contains the handling hole as quickly as possible. The deep learning method is selected according to the above analyses. At present, numerous methods have been proposed for object detection, in which the YOLO network has a high efficiency and can be used in a real-time detection.

The YOLO-V3 network [20] is the latest version of YOLO and has achieved a trade-off between accuracy and real-time performance. Darknet-53 is used as a backbone network of the YOLO-V3 model and consists of 53 convolutional layers. The residual module is introduced into the Darknet-53 network, which helps overcome the gradient problem of a deep network. In addition, a multi-scale prediction and bounding box regression are also added, which makes YOLO-V3 more effective and accurate than YOLO-V2.

**Figure 7:**
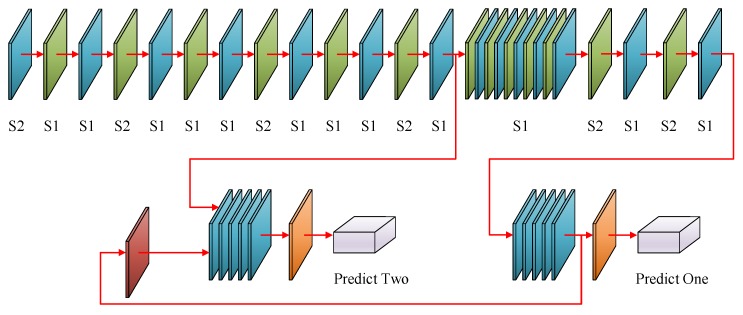
Structure of the YOLO-MobileNet.

**Figure 8:**
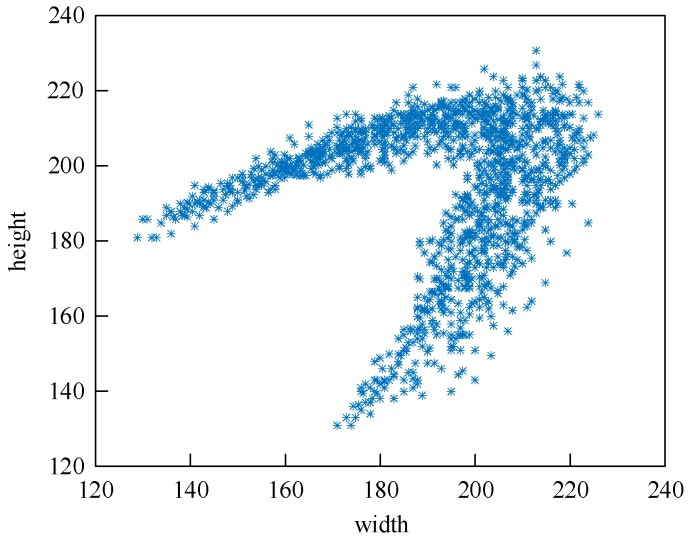
The distribution of handling hole image set.

To meet the requirements of this study, the raw YOLO-V3 model should be modified, and an improved version which could be called YOLO-MobileNet is employed. Figure 7 shows the network structure of YOLO-MobileNet, and the following two points should be illustrated:

(1) There is only one object to detect in this study. To improve the efficiency, MobileNet [26] is used instead of darknet-53 as the backbone. MobileNet is a lightweight network proposed by Google, which stacks several layers of depthwise separable convolutions. By weighing the delay time and accuracy requirements, a MobileNet architecture of the right size and speed is built based on the width and resolution factors. The basic idea of its network structure is to completely separate the correlation and spatial correlation between channels, and significantly reduce the number of calculations and parameters.

(2)Figure 8 shows the size distribution of the bounding boxes. The coordinates *x* and *y* indicate the width and height of the ground-truth bounding boxes, and each blue * indicates one instance. It could be seen that all points are concentrated in a V-shaped region and there are no small objects because almost all of the sizes are larger than 120 pixels. Therefore, the number of predictions is reduced to two and the number of anchors is reduced to six. The K-means method is then used to obtain six clusters from all points, and the results are used to set the anchor size.

In theory, the output of the trained YOLO-MobileNet model is always the ROI of the handing hole. In fact, the detection error cannot be zero owing to the limitations of the deep learning method itself. When a detection error occurs, the output of the subsequent step must be wrong. To ensure the reliability of the locating system, an examination is established here, and the following three terms are added:

(1) The number of objects is one.

(2) The area of the predicted bounding box is within a reasonable range, which is obtained from Figure 8.

(3) Assume that the area of the predicted bounding box of the (i−1)-th image is $S_{i-1}$. The area of the predicted bounding box of the i-th image should be in $\left[S_{i}-\zeta ,S_{i}+\zeta \right]$, where ζ is a threshold.

The results satisfying the above three terms can only be considered as a correct output of the YOLO-MobileNet model. In this way, the location error caused by the YOLO-MobileNet model can be significantly reduced.

### 4.2. Accurate Location of Handling Hole Based on Hough Transformation

**Figure 9:**
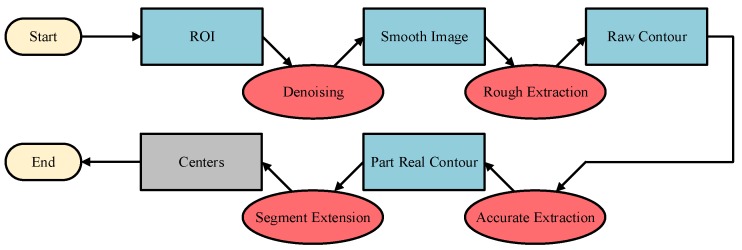
Flowchart of the accurate location.

The most effective way to locate a handling hole is to find the four corners because the handling hole is a rectangle. However, it can be seen from Figure 4a that these contour corner points are not obvious in the presence of dust. It is therefore difficult to extract the corner points directly. Fortunately, a portion of the contour is still visible and the handling hole still looks like a rectangle. If the clear part of contour can be extracted, the intersection points where the contour extend can be regarded as the corner points. The location method is illustrated in detail below, and a flow chart is shown in Figure 9.

**Figure 10:**
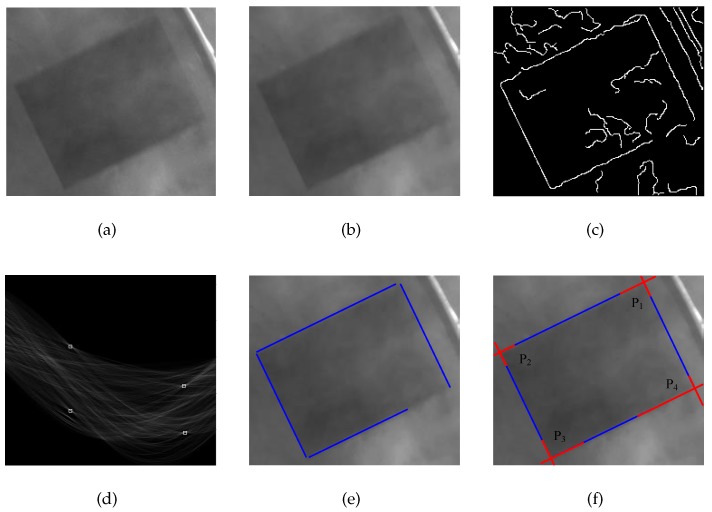
Processing of accurate handing hole location: (**a**) ROI, (**b**) denoised image, (**c**) result of counter detection, (**d**) parameter coordinate system, (**e**) raw line detection result, (**f**) result of corner extraction.

First, we should expand the ROI, which is the result of a rough location. It is necessary to ensure that the handing hole is contained in the ROI completely because the purpose is to extract the contour of the handing hole. This requires an accurate output of the network, which is difficult to achieve. To this end, the ROI expansion is proposed to optimize the ROI to achieve this goal. The ROI in the image is usually shown as a vector $\left(x,y,l_{width},l_{height}\right)$, where (x,y) is the coordinate of the top-left corner and $l_{width}$ and $l_{height}$ are the width and height of the bounding box. Assuming that the expansion length in each direction is $l_{exp}$, the vector of the expanded ROI can be shown as $\left(x-l_{exp},y-l_{exp},l_{width}+2l_{exp},l_{height}+2l_{exp}\right)$. Here, $l_{exp}$ is related to the accuracy of the network and the result is shown in Figure 10a.

A filtering is then needed to remove noise in the image. By observing Figure 4a, the dust in the image can be regarded as additive noise, which makes the counter of the handing hole appear unclear and the inside of the handing hole looks unsmooth, which is similar to the effect of Gaussian noise. Therefore, an image filter is needed, and the counter cannot be blurred after filtering. Although a bilateral filter can achieve this goal, its time consumption is unsatisfactory owing to the large number of computations. Therefore, we conduct multiple morphological operations to improve the quality of the ROI. The corresponding corrosion and expansion operators can be defined as follows:

(4) $$\left[I⊖S_{a}\right]\left(x,y\right)=\min_{s,t\in S}\left\{I\left(x+s,y+t\right)\right\},$$

(5) $$\left[I⊕S_{a}\right]\left(x,y\right)=\max_{s,t\in S}\left\{I\left(x+s,y+t\right)\right\},$$

where $S_{a}$ is an a×a mask. The ROI after the operations is $\overset{˚}{I}$, and thus $\overset{˚}{I}=I_{0}⊖S_{0}⊕S_{0}⊖S_{1}⊕S_{1}\ldots ⊖S_{n}⊕S_{n}$. We can see from Figure 10b that, after several corrosion and expansion operations, the ROI is smoother and the counter of handling hole remains clear.

In the next step, the canny method is used to find all points that might be on the counter. The Canny method is one of the most commonly used edge detection methods and its popularity could be attributed to its optimality [27]. It should be noted that, not only are the true points found in the results, some other points can also be seen, as shown in Figure 10c. The reason for this is that the edge detection can be regarded as a specific type of image segmentation, which can be defined as follows:

(6) $$\tilde{I}\left(x,y\right)=\left\{\begin{array}{cc} counter & I\left(x,y\right)⋈\tilde{\Psi }\left(I\left(x,y\right)\right),\\ background & other, \end{array}\right.$$

where $\tilde{\Psi }$ represents a series of operations, and ⋈ indicates that point I(x,y) can set up $\tilde{\Psi }$. In addition, $\tilde{\Psi }$ of the canny method has four steps: denoising, a gradient calculation, non-maximum suppression (NMS), and false edge removal. The points that meet $\tilde{\Psi }$ are considered along the contour. Moreover, the first step could be abandoned in this study because the image had already been filtered.

**Figure 11:**
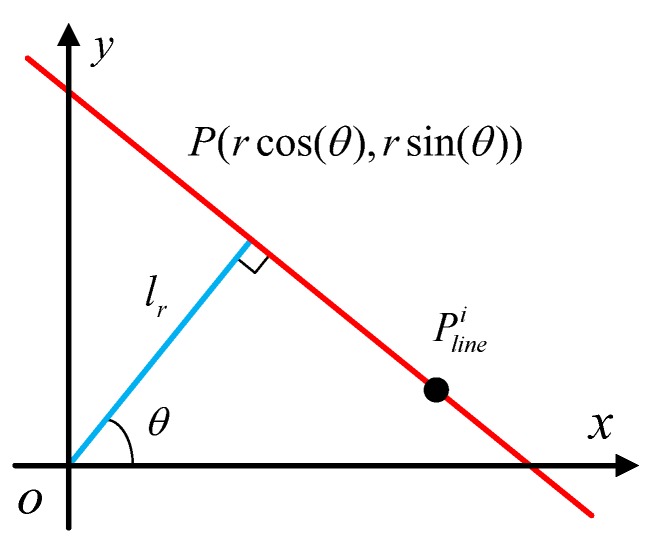
Schematic of a line.

The next step is to perform a Hough transformation to find the real counter from Figure 10f. Compared with other methods, it is easy to implement and has strong strong robustness [28]. As shown in Figure 11, for any point $P_{line}^{i}\left(x,y\right)$ on the line *L*, Equation (7)is set up:

(7) $$\frac{r\sin\theta -y}{r\cos\theta -x}=-\frac{1}{\tan\theta },$$

where $l_{r}$ is a line through which the origin is perpendicular to *L*. The length of $l_{r}$ is equal to *r*, and the angle between $l_{r}$ and the *x* direction is θ. Thus, Equation (7) can be rewritten as follows:

(8) r=xcosθ+ysinθ.

For any point in the Cartesian coordinate, there must be a curve in the parameter coordinate and a mapping between the Cartesian coordinates, and the parameter coordinate is therefore established. We then calculate all lines corresponding to all white points in Figure 10c, and a voting system is established to count the number of lines passing through each point in the parameter coordinate. The statistical results are shown in Figure 10d, and there are four points $\left(r_{i},\theta_{i}\right)$ in which the highest number of votes are marked, where i=1,2,3,4. To obtain the real counter in the Cartesian coordinates, we substitute $\left(r_{i},\theta_{i}\right)$ into Equation (8), the result of which is shown in Figure 10e.

To ensure the reliability of the location method, another examination is added here. The following two terms are applied:

(1) The number of lines obtained by a Hough transformation is greater than three.

(2) All lines are classified according to the slope, and there must be two lines with a large intercept in each class.

If the above terms are met, the detection results are regarded as correct, and the following step should be conducted. Otherwise, these images will be abandoned and the system will continue to process the next image.

Finally, we should extract all corners. As shown in Figure 10e, the four blue lines are only a part of the counter. To obtain all corners, all blue lines should be extended, and the four intersection points can be regarded as the corners, as shown in Figure 10f. In this way, the accurate location of the handing hole is achieved using the above methods.

### 4.3. 3D Reconstruction of Handing Hole

**Figure 12:**
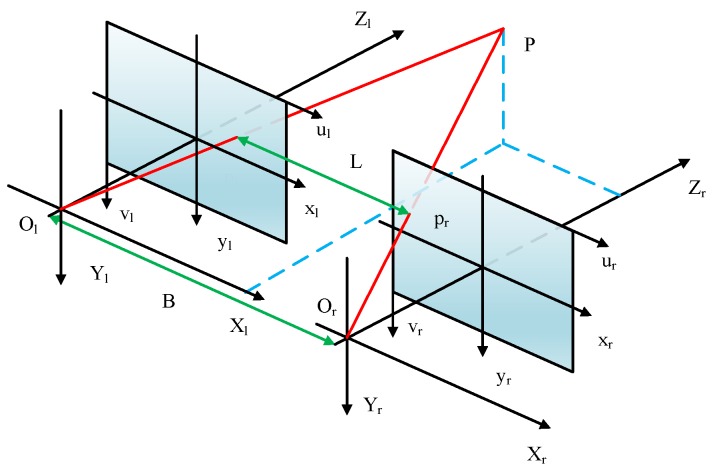
Principle of binocular stereo vision.

The pose and position of a handing hole is obtained by establishing a 3D model. Figure 12 shows the principle of binocular stereo vision. The point *P* in the left and right images are $P_{l}$ and $P_{r}$, respectively. In addition, *B* is the baseline, which can be obtained through a calibration, and *L* is the disparity calculated through stereo matching.

Then, the *z*-coordinate of point *P* will be calculated according to the triangle similarity, and the 3D coordinate in the left camera coordinate will be calculated as follows:

(9) $$x=\frac{x_{l}*b}{x_{l}-x_{r}},z=\frac{b*f}{x_{l}-x_{r}},y=\frac{b*y}{x_{l}-x_{r}}.$$

**Figure 13:**
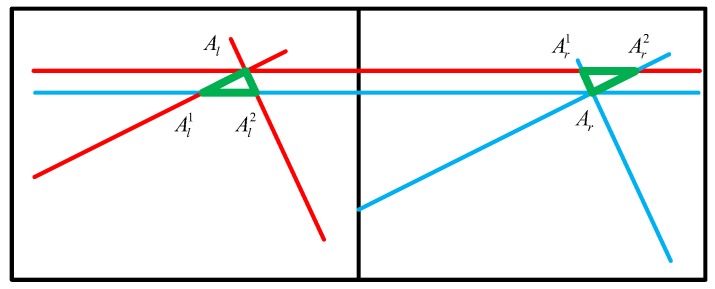
Schematic of non-collinear problem for matching points.

Another problem also needs to be solved. Figure 13 shows a non-collinear problem of the matching points. Here, $A_{l}$ and $A_{r}$ are a pair of matching points, which are not on the same line. In this case, a larger reconstruction error can occur when using the result of Equation (9) directly. As the reason for this, the corner points on both the left and right are obtained by contour fitting rather than stereo matching. The error caused by this case can be called a non-collinear error. Here, point *A* is regarded as an example to illustrate a new method for reducing non-collinear errors.

(1) Calculate the difference between $y_{P}^{l}$ and $y_{P}^{r}$. The reconstruction should be conducted without the following steps when the result is less than threshold $E_{P}^{rl}$. Otherwise, the second step is applied.

(2) Create a line $l_{l}$ through point $A_{l}$ and another line $l_{r}$ through point $A_{r}$. The slope of these two lines is zero. In the left image, two intersection points $A_{l}^{1}$ and $A_{l}^{2}$ will be calculated and a triangle whose points are $A_{l}^{1}$, $A_{l}^{2}$, and $A_{l}$ will be established. In the same way, another triangle is established in the right image.

(3) The centers of the two triangles are calculated and will replace $A_{l}$ and $A_{r}$ as the new corners.

Each point should be processed by the above method, and perfect matches will then be obtained.

Assuming that the robot coordinate is regarded as a reference, an extra step of coordinate transformation is then needed. This process can be expressed through Equation (10):

(10) $$\left[\begin{array}{c} X_{r}\\ Y_{r}\\ Z_{r}\\ 1 \end{array}\right]=\left[\begin{array}{cccc} m_{11} & m_{12} & m_{13} & m_{14}\\ m_{21} & m_{22} & m_{23} & m_{24}\\ m_{31} & m_{32} & m_{33} & m_{34}\\ 0 & 0 & 0 & 1 \end{array}\right]\left[\begin{array}{c} X_{c}\\ Y_{c}\\ Z_{c}\\ 1 \end{array}\right],$$

where *M* is the transform matrix obtained through a hand-eye calibration. In addition, $\left(X_{r},Y_{r},Z_{r}\right)$ are the robot coordinates and $\left(X_{c},Y_{c},Z_{c}\right)$ are the camera coordinates. The 3D model will then be established using the four corner coordinates. Next, we calculate the position and pose of the handing hole. To reduce the measurement error, $\left(\tilde{x},\tilde{y}\right)$ calculated using Equation (11) can be regarded as the center point, and its coordinate is used as the position:

(11) $$\tilde{x}=\sum_{n=1}^{4}x_{i},\tilde{y}=\sum_{n=1}^{4}y_{i}.$$

To calculate the pose, a vector $\vec{EF}$ based on four corners should be set up. The center of the edge closer to the profile of the reactor is treated as the starting point *E*, and the center of the edge away from the rector profile is regarded as ending point *F*. The goal is to make the vector EF parallel to the plane XoZ and the angle between $\vec{EF}$ and $\vec{e_{z}}\left(0,0,1\right)$ less than 90°. Therefore, the angle between EF and $\vec{e_{y}}\left(0,0,1\right)$ and $\vec{e_{z}}\left(0,0,1\right)$ is as expressed through Equation (12):

(12) $$\theta_{y}=\arccos\frac{\vec{EF}\times \vec{e_{y}}}{\left|\vec{EF}\right|\left|\vec{e_{y}}\right|},\theta_{z}=\arccos\frac{\vec{EF}\times \vec{e_{z}}}{\left|\vec{EF}\right|\left|\vec{e_{z}}\right|}.$$

## 5. Experiments and Discussion

### 5.1. Data Acquisition and Annotation

**Figure 14:**
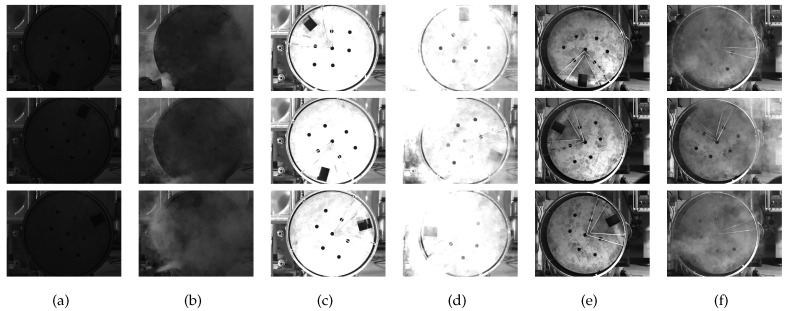
The categories of training images: (**a**) low brightness images without dust, (**b**) low brightness images with dust, (**c**) high brightness images without dust, (**d**) low brightness images with dust, (**e**) normal brightness images without dust, (**f**) normal brightness images with dust.

Whether the network can process the images collected in different cases depends on the integrity of the dataset [29]. To enhance the richness of the dataset effectively, the image set contains six types of images, as shown in Figure 14. There are 1800 images in the dataset. All images are collected on site, and the dataset augmentation is not needed because we have complete experiment equipment for simulating all situations occurring during production.

The following two points should be introduced:

(1) To obtain the images of the handling hole at different angles, the model is rotated during the acquisition.

(2) To simulate different illuminations, we adjust the aperture and brightness of the external light source.

(3) The dust concentration is an uncontrollable factor. The zinc powder has difficulty remaining in the air for a long time owing to its large density. We use a smoke generator instead of artificial dusting. We inject smoke into the air at intervals, and 500 images will then be obtained as a raw dataset in each illumination. We manually select 300 images to add to the image dataset.

In the next moment, LabelImg is used to label all images. To improve the accuracy of the network, we should try to make the rectangle containing only the handling hole. Finally, we divide the image dataset. The training set has 1260 images, which are randomly selected from the image set. There are 360 images randomly selected to form the new test set, and the rest of the images form the validation set. The percentages of the training set, test set, and validation set are approximately 70%, 20%, and 10%, respectively.

### 5.2. Model Training

**Figure 15:**
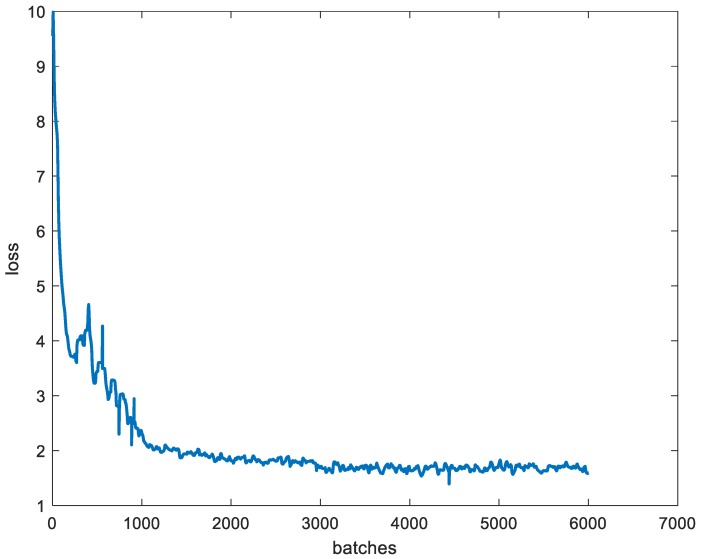
The training loss curve.

The models are trained and tested on a computer with a Nvidia RTX2080. Considering the memory constraint of the GPU, the batch size is no more than 6. The momentum is set to 0.9, the learning rate is set to 0.001, and the decay is set to 0.0005. It should be noted that there is only one object and the dataset is not large, and thus the epoch cannot be set too big, or an over-fitting will occur. In this case, the epoch is set to 6000. The model is then trained after defining the training parameters. Figure 15 shows the training loss curve. It can be seen that the initial value is large and the training loss then decreases rapidly during the first 1000 batches. As the number of training batches increases, the loss slowly decreases and gradually tends to reach stabilization. The loss value fluctuates at around 2 after 3000 batches, which is the ideal result. Excessive training may lead to an over-fitting, in the training model, the weight is output every 100 batches, and we need to evaluate all the results to choose the best weight.

### 5.3. Evaluation

In this section, we describe the testing of the RAL method under different conditions to evaluate if it can be used in an industrial environment.

#### 5.3.1. Preference of YOLO-MobileNet

We tested the performances of YOLO-MobileNet and the complete RAL method. It should be noted that, in several studies, the detection results of partial occlusion objects, multiple objects, and small objects are usually regarded as a reference to evaluate a network. Such cases will not occur during the sherardizing process. Even if the handing hole is blocked completely by dust, a secondary inspection will be used to maintain the reliability of the system. Therefore, in this study, we only need to estimate whether YOLO-MobileNet can find the ROI of the handing hole in a general industrial environment.

Table 1 lists the test results of YOLO-MobileNet using several categories of images. Table 2 shows a comparison with four other object detection models including YOLOv3, SSD, Faster-RCNN, and tiny-YOLOV3, by using the AP parameters and time consumption per image. The AP is higher in the absence of dust and can reach 100%. The detection results of images with dust are worse than those of clear images, and the AP is 91.48%.

**Figure 16:**
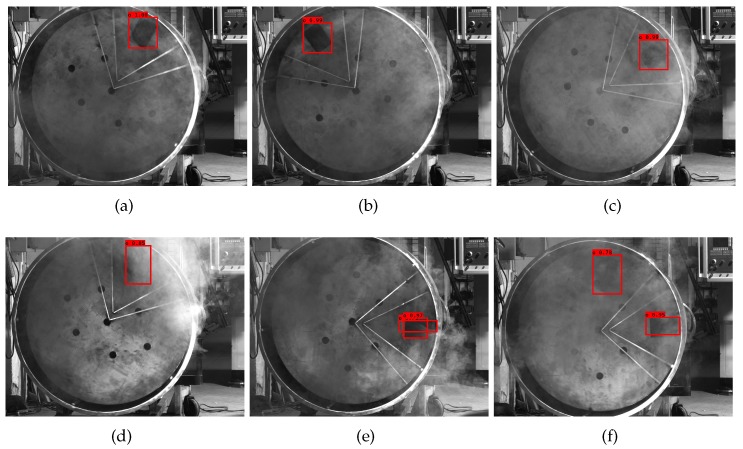
Detection results of YOLO-MobileNet model: (**a–c**) good detection results, (**d–f**) parameter coordinate system.

Some detection results are shown in Figure 16. Figure 16a–d shows good detection results, and Figure 16e–f shows poor detection results. In the environment without dust, there are two main types of detection errors. In the first type, multiple objects are detected. In this case, YOLO-MobileNet not only finds a true object, but it also regards a fake object as the detection result or marks several bounding boxes on the true object. In the second type, the IOU of the detection result is too small. This case occurs when the dust concentration is high, and only one part of the object is contained in the smaller prediction bounding box. In fact, this case rarely occurs because there are some dust removers above the furnace and the dust concentration will not be too large. Even if some unwelcome situations occur, there are two examinations in the ARL method to maintain the reliability of the locating system.

#### 5.3.2. Preference of RAL Method

The program is applied using an Intel(R) Core(TM)i7-8700 CPU running at 3.30 GHz with 16 GB of RAM. The GPU is a Nvidia RTX2080. To reduce the time consumption, a multithreading method is used to process the left and right images, and, when the accurate location of two images is completed, the position and pose calculation are applied.

The average process time of YOLO-MobileNet is 0.019 s is shown in Table 2, and the time consumption of other steps is shown in Table 3. The average time required for the RAL method to process a pair images is 0.23 s, which means that there is sufficient time remaining for the motion control system and a large lag will not occur.

**Figure 17:**
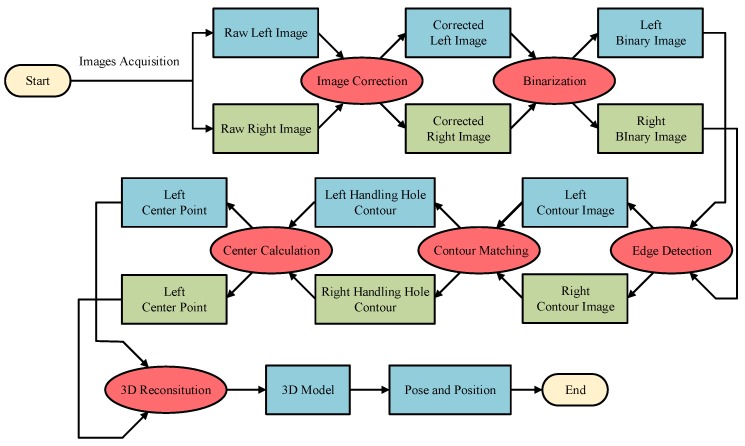
Flowchart of the true value calculation.

The location error refers to the distance between the measured and true values. The smaller the error, the higher the location precision. However, we only have a model and a robot has yet to be installed, and thus it is difficult to evaluate the precision of the RAL method because we are unable to obtain the true value dynamically. A new static evaluation method is proposed to evaluate the precision of the RAL method. Figure 17 shows a flow chart. First, the reactor is set at an angle and maximizes the difference between the handing hole and the surrounding by adjusting the auxiliary light and CCD aperture. Next, the threshold segmentation method is used to obtain the perfect counter of the handing hole, and the four corners are manually obtained. Finally, a 3D model will be established and the location information of the handing hole can then be easily calculated. Therefore, a static location result obtained by the above method in a smokeless environment can be approximated as a true value.

We then eject the smoke in the front of the rector, and the result obtained by the RAL method is regarded as the measured value. By repeating the above process, there are several real values $\zeta_{r}^{i}$ and the measured value $\zeta_{m}^{i}$ will be obtained. The measurement error $\zeta_{e}^{i}$ is equal to $|\zeta_{m}^{i}-\zeta_{m}^{i}|$ and the average measurement error $\zeta_{e}^{n}$ is then defined as follows:

(13) $$\zeta_{e}^{n}=\frac{1}{n}\sum_{i=1}^{n}\zeta_{e}^{i}.$$

**Figure 18:**
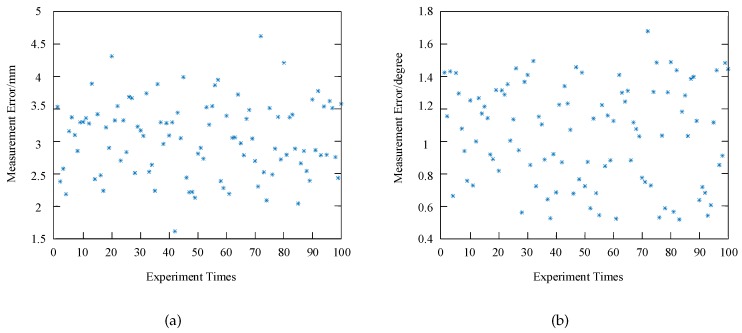
Measurement error in the experiment: (**a**) Measurement error of distance, (**b**) Measurement error of rotation angle.

There are 100 sets of measurement results shown in Figure 18. It can be seen that the maximum measurement error is less than 1.68° and 4.62 mm, which indicates that the RAL method achieves a high accuracy.

### 5.4. Encapsulation

**Figure 19:**
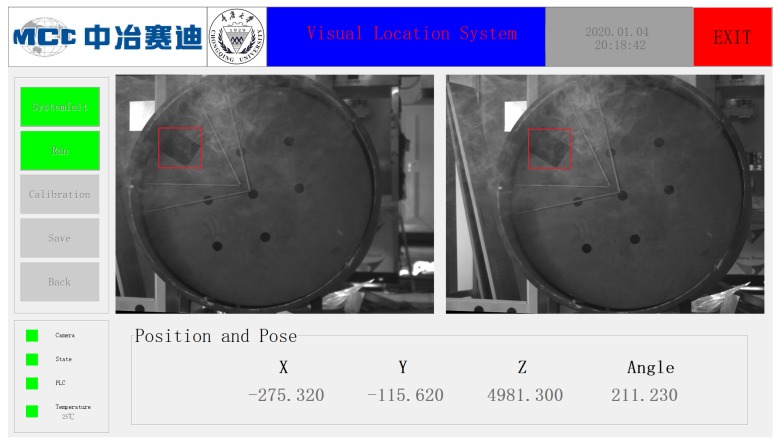
Interface of software.

To achieve real-time monitoring and a real-time output of the measured values, we need to package the entire system into a single software, which is coded using C++ with OpenCV 3.2, and the operational interface is coded using MFC. The interface of the software is shown in Figure 19. The software consists of three parts: (1) a calibration system, (2) a real-time display system consisting of an image display and an information display, and (3) an information storage system designed to save important data during a program operation.

## 6. Conclusions

To achieve an automatic unloading of the reactors during the sherardizing process, in this study, the defects of the existing method were first analyzed. Next, a RAL method was presented, aiming to calculate the coordinate and angle of the handing hole. This method is divided into three steps. First, the ROI of the handing-hole is obtained using an improved version of YOLO-MobileNet. This model is more suitable for positioning holes than other versions of the YOLO model. Second, a precise positioning method consisting of a series of conventional image processing algorithms is proposed, which can find the four corners of the hole. Finally, the pose and position of the handing hole is calculated by establishing a 3D model. The experiment results show that the the measurement error of the RAL method is less than 4.64 mm and 1.68° and the average measurement time for a pair of images is approximately 0.21 s, which can meet the requirements of visual locating. 

## Figures and Tables

**Table 1:** Test results of YOLO-MobileNet.

Parameters	Image Category					
	Dark	Dark&dust	Bright	Bright&dust	Normal	Normal&dust
AP-50	88.48%	82.48%	90.16%	88.28%	92.65%	90.53%
AIOU	0.81	0.79	0.83	0.8	0.86	0.83

**Table 2:** Comparison with four popular object detection models.

Model	Time(s)	AP(%)
YOLO-MobileNet	0.02	90.53%
SSD	0.15	82.21%
Faster-RCNN	0.12	87.35%
YOLOV3	0.07	96.57%
tiny-YOLOV3	0.02	88.31%

**Table 3:** Time consumption of each step.

Step	Time Consumption (s)
ROI expansion	0.002
Denoising	0.05
Counter detection	0.15
Corners extraction	0.01
All	0.21

## Abbreviations

The following abbreviations are used in this manuscript:

IPC	Industrial personal computer
PCI	Peripheral component interconnect
CCD	Charge coupled device
YOLO	You only look once
SSD	Single shot multiBox detector
MBB	Minimum bounding box
IoU	Intersection over union
RAL	Rough-accurate location
ROI	Region of interest
CPU	Central processing unit
GPU	Graphics processing unit
